# Improved Image Fusion in PET/CT Using Hybrid Image Reconstruction and Super-Resolution

**DOI:** 10.1155/2007/46846

**Published:** 2006-12-24

**Authors:** John A. Kennedy, Ora Israel, Alex Frenkel, Rachel Bar-Shalom, Haim Azhari

**Affiliations:** ^1^Faculty of Biomedical Engineering, Technion – Israel Institute of Technology, Haifa 32000, Israel; ^2^Department of Nuclear Medicine, Rambam Health Care Campus, Haifa 35245, Israel; ^3^The Ruth and Bruce Rappaport Faculty of Medicine, Technion – Israel Institute of Technology, Efron Street 1, P.O. Box 9649 Bat Galim, Haifa 31096, Israel

## Abstract

*Purpose*. To provide PET/CT image fusion with an improved PET resolution and better contrast ratios than standard reconstructions. *Method*. Using a super-resolution algorithm, several PET acquisitions were combined to improve the resolution. In addition, functional PET data was smoothed with a hybrid computed tomography algorithm (HCT), in which anatomical edge information taken from the CT was employed to retain sharper edges. The combined HCT and super-resolution technique were evaluated in phantom and patient studies using a clinical PET scanner. *Results*. In the phantom studies, 3 mm^18^F-FDG sources were resolved. PET contrast ratios improved (average: 54%, range: 45%–69%) relative to the standard reconstructions. In the patient study, target-to-background ratios also improved (average: 34%, range: 17%–47%). Given corresponding anatomical borders, sharper edges were depicted. *Conclusion*. A new method incorporating super-resolution and HCT for fusing PET and CT images has been developed and shown to provide higher-resolution metabolic images.

## 1. INTRODUCTION

Positron emission tomography (PET) provides images of metabolic processes that are used increasingly in the clinical setting to obtain data on cancer and other pathological processes. In oncology, the diagnosis of cancer and the assessment of the extent of disease often rely on PET [1]. However, because PET images are relatively noisy and are limited by relatively poor spatial resolution, small lesions are difficult to detect [2] and the anatomical location of hypermetabolic regions can be difficult to determine in PET images [3].

The introduction of dual modality PET/CT scanners [4, 5] has addressed the latter issue by providing metabolic PET images registered with the anatomical information from CT. In these scanners, the patient lies still on a bed which is then translated through fixed mechanically aligned coaxial CT and PET gantries so that the data acquired are precisely coregistered in space. The PET acquisition typically occurs immediately after the CT acquisition to minimize the effects of patient motion. After reconstruction, the high-resolution anatomical images (from CT) are overlayed with the functional images (from PET) to provide precise localization of hypermetabolic regions. In oncology, such image fusion has been shown to improve the diagnostic reliability [6, 7].

**Figure 1:**
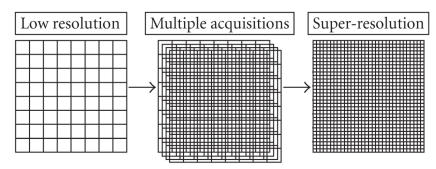
Super-resolution algorithms combine multiple low-resolution image acquisitions into a high-resolution image.

In the interest of improving small lesion detectability, the objective of this study was to provide a new method for PET/CT image fusion with an improved resolution and better contrast ratio relative to standard reconstructions. First, a modified form of the super-resolution method of Irani and Peleg [8] shown to improve resolution in PET imaging (Kennedy et al. [9]) was employed for PET data acquisition and image reconstruction. In the super-resolution method, several acquisitions interspersed with subpixel shifts are combined in an iterative algorithm to yield a higher-resolution image, depicted schematically in Figure 1. Secondly, since the radiopharmaceutical distribution will often follow anatomical borders, the potential exists to combine the high-resolution border information from the CT image with the functional distribution from the PET image to yield a PET image with enhanced borders. The algorithm we used to incorporate CT data in PET images is called hybrid computed tomography (HCT). HCT was originally developed for artifact reduction in ultrasonic computed tomography [10]. In regions not containing anatomical edges, HCT has been shown to provide noise reduction in PET images equivalent to the standard Gaussian filtering typically used [11]. In PET imaging, HCT provides sharper edges and improves contrast ratios [11].

In this paper, we demonstrate how a combination of a super-resolution acquisition and reconstruction combined with HCT filtering increases the contrast ratios of ^18^F-FDG uptake in PET images while providing noise reduction equivalent to a standard Gaussian filter in regions without corresponding anatomical edges. Where corresponding anatomical edges are available, the technique enhances the edges of ^18^F-FDG uptake. Through the combination of increased resolution and edge enhancement, the PET imaging of small features is improved.

## 2. MATERIALS AND METHODS

PET was performed using standard and super-resolution acquisitions [9]. Each type of acquisition was then filtered with one of two techniques: a standard Gaussian filter or the equivalent HCT filter [11] incorporating CT border information. Consequently, four methods of generating PET images were compared:

- standard acquisition and processing with Gaussian filtering;
- super-resolution acquisition and processing with Gaussian filtering;
- standard acquisition and processing with HCT filtering;
- super-resolution acquisition and processing with HCT filtering.

The degree of filtering was chosen to keep the level of noise constant among images compared.

### 2.1. Super-resolution and HCT

The term super-resolution refers here to a technique in which several low-resolution points of view (POVs) are combined iteratively to obtain a higher-resolution image. In the Irani and Peleg formulation of a super-resolution algorithm [8], the initial estimate of the high-resolution image, *f* ^(0)^, can be based on the average of the upsampled acquisitions shifted to a common reference frame:

(1) $$f^{\left(0\right)}=\frac{1}{K}\sum_{k=1}^{K}T_{k}^{-1}\left(g_{k}↑s\right),$$

where *g_k_* is one of *K* acquisitions, *T* ^−1^_*k*_ is the geometric transformation to a common reference frame, and ↑ *s* is the upsampling operator from low-resolution to the high-resolution representation.

One could obtain the low-resolution measured data *g_k_* from the “true” image *f* if the acquisition system was adequately modeled. The process would include shifting the image *f* to the *k*th POV, blurring to account for limited system resolution, downsampling to the system's sampling rate, and adding noise. For a given estimate of the image, *f* ^(*n*)^, the low-resolution data is modeled as in [8]:

(2) $${\tilde{g}}_{k}^{\left(n\right)}=\left(T_{k}\left(f^{\left(n\right)}\right)*h\right)↓s,$$

where ∗*h* is the blurring operation with the kernel *h* and *s* ↓ is the downsampling operator which averages the pixels to the lower resolution. The noise term is dropped. The original geometric transformation of the *k*th acquisition from the common reference frame is *T_k_*. This is typically the physical shift between the object and the imager from the original position.

To obtain a better estimate of the image *f*, the previous estimate of the high-resolution image, *f* ^(*n*)^, is corrected by the difference between the low-resolution data *g_k_* and the term ${\tilde{g}}_{k}^{\left(n\right)}$ that represents what the low-resolution data would have been, had the estimate, *f* ^(*n*)^, been correct. The next iteration *f* ^(*n*+1)^ of a high-resolution estimate is the following [8]:

(3) $$f^{\left(n+1\right)}=f^{\left(n\right)}+\frac{1}{K}\sum_{k=1}^{K}T_{k}^{-1}\left(\left(\left(g_{k}-{\tilde{g}}_{k}^{\left(n\right)}\right)↑s\right)*p\right).$$

Here, the differences between *g_k_* and ${\tilde{g}}_{k}^{\left(n\right)}$ are upsampled, ↑ *s*, to achieve the smaller super-resolution pixel size, moved to a common reference frame, *T* ^−1^_*k*_, and averaged over *K* acquisitions. The symbol ∗*p* is a sharpening kernel. This formulation of the super-resolution algorithm has been demonstrated to improve resolution in MRI imaging [12, 13] and in PET [9].

Although the blur and sharpening kernels can be set to unity [9, 12], in this study the blur kernel has been modeled as a Gaussian point spread function (PSF). In order to reduce the noise caused by sharpening, the upsampling procedure of Farsiu et al. [14] was used.

In addition to the super-resolution acquisition, a modified form of an iterative algorithm called hybrid computed tomography (HCT), implemented previously on ultrasonic CT data [10], was utilized here to fuse CT anatomical data with the PET functional data. The HCT algorithm is based on a two-dimensional (2D) Taylor series expansion of the gray levels which incorporates texture and edge information. The HCT algorithm utilizes edge information taken from CT to retain sharper edges while smoothing the PET data, which often follow the anatomical borders. Thus, the resulting reconstructed image has reduced noise but sharp borders.

**Figure 2:**
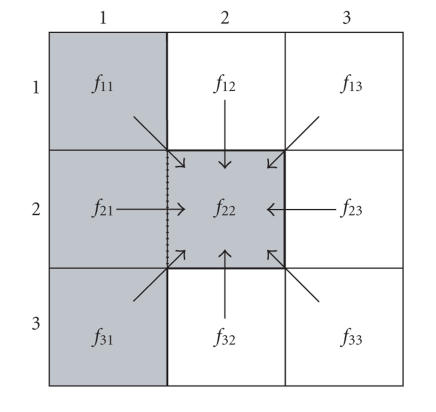
HCT applied to a 3 × 3 image. In the case that pixel *f*_22_ indicates a true uptake (gray), the solid line is the true border and HCT algorithm iteratively averages its value with the pixels in the first column. In the case that dotted line is the true border, the uptake in pixel *f*_22_ iteratively averages its value with the pixels in the second and third columns.

In HCT, each value of the image *f* at each pixel is expanded into neighboring pixels. Neglecting higher-order terms, the modified 2D Taylor expansion about pixel (*a, b*) has a value *f* (*x, y*) at pixel (*x, y*) [10]:

(4) $$f\left(x,y\right)=f\left(a,b\right)+\left[\left(x-a\right)\cdot \frac{\partial f}{\partial x}{|}_{a,b}+\left(y-b\right)\cdot \frac{\partial f}{\partial y}{|}_{a,b}\right]\cdot \beta \left(a,b\right),$$

where the function *β*(*x, y*) has a zero value within homogeneous regions but is set to have a value of 1 at boundary points. In the PET/CT application, the function *β* can be obtained from the anatomical edge data of the CT scan. One method of modifying (4) to include discrete pixels and diagonal directions is to write it as

(5) $$f\left(x,y\right)=f\left(a,b\right)+\left[{\text{Δ}}_{r}\cdot {\frac{\text{Δ}f}{{\text{Δ}}_{r}}|}_{a,b}\right]\cdot \beta \left(a,b\right),$$

where Δ*_r_* is the step size in the direction $\vec{r}=\left[x-a\;\;y-b\right]$ and Δ*f* = *f* (*x, y*) − *f* (*a, b*). Here, the expansion was limited to nearest neighbors, as depicted in Figure 2, so the step size was unity: Δ_*r*_ = 1. In one HCT iteration, (5) is applied in a neighborhood of *f* (*x, y*) and the results averaged, for each pixel (*x, y*) in the image. In the absence of a border, repeated iterations of (5) average a pixel value with its neighbors. If a 3 × 3 neighborhood is used, in regions distant from a border, it can be shown that *n* HCT iterations are equivalent to the application of a Gaussian filter with a full-width half-maximum (FWHM) of [11]:

(6) $$\text{FWHM}=4\sqrt{\frac{\ln\left(2\right)n}{3}}\text{pixels}.$$

If the functional and anatomical boundaries do not match, HCT may introduce artifacts [11], but in the absence of border information the default of HCT is the standard Gaussian filtering.

For a simple HCT example, consider the 3 × 3 image in Figure 2. The central pixel *f*_22_ has an uptake indicated by the gray shading. In the first HCT iteration, the value assigned to *f*_22_ by (5) is determined by its nearest neighbors. If the thick solid line is the true border, *β* between the central pixel and the 3 gray pixels in the first column is set to 0 because there is no border among them and (5) sets the value of *f* (*x, y*) to *f* (*a, b*). However, when the index (*a, b*) falls on the other side of the border, *β* is set to 1 and *f* (*x, y*) retains its original value. When applied to all 9 neighborhood pixels, the uptake in the central pixel is averaged with the uptake in those 3 gray pixels in the first column. Equation (5) generates a weighted average; in this case the center pixel is weighted at 6/9 and the 3 other pixels are weighted at 1/9 each. However, if the true border is between the central pixel *f*_22_ and *f*_12_, as indicated by the dotted line, then *β* is set to 0 only among the pixels of the second and third columns. In the first iteration, the value of the central pixel is averaged with the 5 other pixels in the second and third columns which have no uptake (as indicated by white). Although the value of the central pixel is substantially reduced, the application of (5) to each of the other 5 pixels in turn effectively distributes this uptake among the 6 pixels in the second and third columns. Regardless of the position of the border, the application of (5) is an averaging operation; therefore HCT is a counts-preserving process.

The combined technique (i.e., super-resolution and HCT) was evaluated in both phantom (3D brain-mode acquisition) and patient studies (2D whole-body mode acquisition), using a clinical PET scanner (GE Discovery LS, GE Healthcare Technologies, Milwaukee, WI).

### 2.2. Data acquisition and processing

The GE Discovery LS combines X-ray CT and PET scanners arranged such that the gantries are coaxial and a bed can automatically move through each gantry in order to provide images in both modalities that are coregistered. The PET portion of the scanner is similar to a GE Advance NXi described elsewhere [9, 15]. In a standard 2D whole-body PET acquisition, the septa between the 18 detector rings restrict the photons acquired to the transaxial plane. Transaxial images (35 per field of view, FOV) are typically reconstructed as 128 × 128 pixel images having a pixel size of 4 mm × 4 mm and a slice thickness of 4.25 mm. The axial FOV is 14.5 cm and the transaxial FOV, as reconstructed in this mode, is 50 cm. An ordered subsets expectation maximization (OSEM) algorithm [16] using 2 iterations and 28 subsets was used for reconstructing the 2D whole-body data from the PET sinograms (projections). Coronal and sagittal images are typically obtained by stacking the images of several axial FOVs into a three-dimensional (3D) data set and reslicing appropriately.

The 3D brain-mode acquisition is similar except that the septa are retracted to increase the number of photons detected. The data was rebinned into transaxial data sets using Fourier rebinning [17] before being reconstructed with an OSEM algorithm using 5 iterations and 32 subsets. The pixel size is typically set to 2 mm × 2 mm reducing the reconstructed transaxial FOV width by a factor of 1/2. The slice thickness remains the same as in the 2D whole-body mode.

The CT provided 512 × 512 pixels transaxial images with a pixel size of 1 mm × 1 mm and a slice thickness of 4.25 mm which were coregistered with the PET images. A tube voltage of 140 kV and current of 90 mA was used. For attenuation corrected (AC) PET images, the CT images also served as the basis for an attenuation map by means of rescaling the Hounsfield units (HU) of the CT to attenuation coefficients appropriate for the higher energy of PET gamma rays [18–21].

In this study, the 2D whole-body mode data was reconstructed with a voxel size of 2 mm × 2 mm × 4.25 mm, similar to the 3D brain-mode acquisition. This gave transaxial PET images of 256 × 256 pixels for the 2D whole-body mode. This was the voxel size for all the standard acquisitions and for each low-resolution POV in the super-resolution acquisition data sets. After processing with the super-resolution technique, the pixel sizes obtained were smaller. When super-resolution was applied in the transaxial plane (see below), the resulting voxel size was 1 mm × 1 mm × 4.25 mm. When super-resolution was applied axially (see below), the resulting voxel size was 2 mm × 2 mm × 1 mm.

Unfiltered image data sets from standard and super-resolution acquisitions were then filtered with either a standard Gaussian filter or an HCT filter which could incorporate edge information while providing equivalent smoothing (6) in regions away from anatomical edges. The smoothing was set to maintain the same level of noise among the images obtained from the four processing methods (see below). In order to make effective use of the resolution of the border information provided by the CT [11], the filtering was applied after the images had been interpolated to a 0.25 mm × 0.25 mm pixel size for the 3D brain-mode PET/CT acquisitions and 0.5 mm × 0.5 mm for the 2D whole-body case using a piecewise cubic Hermite interpolation. The edges were extracted using a Canny edge detector algorithm [22] on CT images to which the scanner protocol's default contrast window had been applied (level: 40 HU, width: 400 HU). For edge extraction, the Gaussian smoothing employed on the CT by the Canny edge detector was 1.2 mm FWHM for the 3D brain-mode PET/CT acquisitions and 3.0 mm FWHM for the 2D whole-body case.

### 2.3. Phantom study

**Figure 3:**
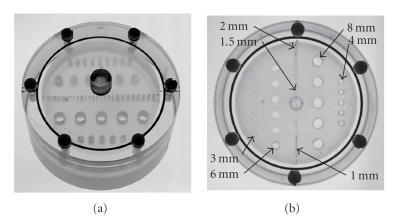
Phantom: a specially treated polycarbonate disk allowed ^18^F-FDG solution to flow freely through holes of varying sizes when immersed in a cup of the solution.

To evaluate image quality among the four processing methods implemented here, a specially designed phantom was used (Figure 3). The phantom provided cylindrical hotspots of ^18^F-FDG solution with diameters of 1, 1.5, 2, 3, 4, 6, and 8 mm arranged in rows such that the separation between hotspots was equal to their diameters. The hotspots were created by drilling holes through a polycarbonate disk (diameter 9 cm, thickness 1.5 cm) and treating the disk with ozone to allow ^18^F-FDG solution (130 kBq/mL) to flow freely through them when the disk was immersed in a fitted cup containing the solution. To a 1 cm depth, on each side of the disk, the cup contained just ^18^F-FDG solution.

**Figure 4:**
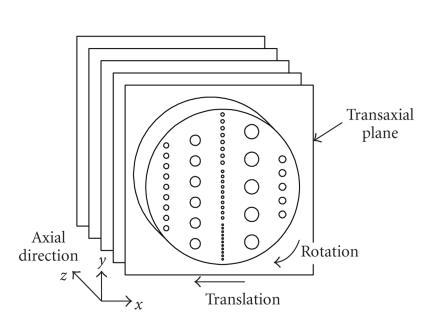
Geometry of phantom orientation for the 3D brain-mode PET acquisition. The phantom disk was aligned with the transaxial plane and translated and rotated within that plane between each of four separate POVs.

The phantom was placed in the scanner to obtain transaxial images in the plane of the disk using the 3D brain-mode acquisition protocol (Figure 4). A standard acquisition of 10-minute duration was followed by 4 acquisitions of 2.5 minutes each for the super-resolution acquisition. Each PET acquisition was accompanied by a CT scan to provide attenuation correction (AC) according to common practice with such PET/CT scanners [18]. Between the 4 acquisitions, the phantom was given a small displacement and rotation in the transaxial plane to provide the geometrical shifts needed by the super-resolution algorithm. The position of the initial acquisition was taken to be the common reference frame. In the case of the phantom trial, the size of the geometric shifts was tracked in the CT images using two 1 mm markers separated by 43 cm that had been fixed to the phantom in the transaxial plane. The shifts used are listed in Table 1. The initial CT image also provided the border information used by the HCT algorithm.

The geometry of the phantom and the method of super-resolution acquisition in the 3D brain mode is described elsewhere [9] in more detail.

For comparison purposes, each processing method was applied to achieve the same degree of noise reduction. As a measure of the noise, the variance in the PET signal was calculated in a region known to have a homogeneous uptake of ^18^F-FDG solution. The transaxial slices of the cup of ^18^F-FDG solution on either side of the polycarbonate disk contained no features except for the 9.0 cm diameter circular edge of the cup. A 5.0 cm diameter circular region of interest (ROI) was selected from one of these slices. Because such a region contains no edges from the CT, both HCT and Gaussian filtering provide the same degree of smoothing [11]. The FWHM (or HCT equivalent) of the smoothing was chosen so that the standard and super-resolution acquisitions and reconstructions had the same variance within this homogeneous ROI. The same filters were then applied to the phantom images containing the features of interest: the uptake in the holes of the polycarbonate disk.

As an indication of image quality, a contrast ratio was calculated for the phantom results. For each row of holes, the locations of the sources were known so they were masked and an average PET signal was calculated. The regions falling between holes were also masked and those pixel values were used to calculate an average background value for that row. The contrast ratio was taken to be the average PET signal to the average background, so that a contrast ratio of unity would indicate that the feature could not be distinguished. Because the level of noise as measured by the variance was kept constant, comparing these contrast ratios was equivalent to comparing a contrast to variance metric.

Three additional studies were performed to measure the PET resolution of this experimental arrangement in terms of a PSF of the data acquisition. A single 1 mm hole of the phantom disk was filled with 20 *μ*Ci (0.74 MBq) ^18^F-FDG solution and capped in order to emulate a “point source” for transverse 3D brain-mode images that were acquired as above. The reference position for the source was 2.0 cm above the axial center line of the scanner. Additionally, to check axial resolution, the phantom was laid flat and fixed to the bed to emulate a “point source” in coronal images. Between each of 4 PET acquisitions, the bed was automatically shifted into the scanner in 1 mm increments, and the super-resolution technique was applied axially. The process was repeated for the 2D whole-body mode. These results have been reported elsewhere [9], but that study used a blurring-and-deblurring kernel of 1 pixel. Here, as a modification, the blur kernel was set to a Gaussian PSF with a FWHM chosen to minimize the FWHM of the “point source” and the blurring-and-deblurring procedure [14] described above was used. For the purpose of direct comparison, the same data set as the previous report [9] was used.

Anticipating the focus of the patient study below, the axial resolution of the 2D whole-body mode was also checked for 2 POVs with 2 mm axial shifts and 8 POVs with 0.5 mm axial shifts.

### 2.4. Patient study

The patient was injected with 370 MBq of ^18^F-FDG after a 4 h fast and was then kept resting comfortably for 90 min before scanning. A 2D head-to-thigh PET/CT scan was acquired, including a CT scan followed by a PET scan consisting of 6 FOVs with an acquisition time of 4 min per FOV. During this standard PET acquisition, the CT was reviewed to identify an ROI suitable for employing the super-resolution technique. A FOV was chosen containing a suspected small lung lesion. After the standard PET scan, the patient was requested to remain still, the bed registration was maintained, and 4 additional POVs of the ROI were acquired, taking 4 min each. Each 4-minute acquisition interval was subdivided into 1-minute and 3-minute intervals so that four 1-minute-long POVs were available to check the case in which the total super-resolution acquisition time equaled the standard acquisition time. Between each subsequent POV, the bed was automatically moved 1 mm further into the scanner to provide 4 PET views differing by shifts which were subpixel since the slice thickness of a standard PET acquisition in the axial direction was 4.25 mm. The patient was not exposed to additional radiation since the X-ray CT scan was not repeated. Because registration was maintained, the initial X-ray CT scan could be used to provide border information for the HCT processing of both the standard and super-resolution PET images by matching the data from any transaxial PET slice with the data from the appropriate transaxial X-ray CT slice at the same location.

As in the phantom trial, the patient images were processed by the four methods. Nonattenuation corrected images were used because the pulmonary lesion was more evident than in the AC PET. The degree of image noise was measured by the variance. In the absence of a known region of homogeneous uptake, the variance was calculated from the nonzero pixel values excluding a 15 mm circular ROI around the lesion of interest in the coronal images. The degree of filtering in each of the four processing methods was chosen to keep the noise level the same, as measured by this variance.

In order to compare PET images in the patient study, target-to-background ratios were calculated as a measure of the intensity of the lesion's uptake for coronal, sagittal, and transverse slices through the lesion of interest. The precise target shape and location were unknown, so the masking method used for the phantom contrast ratio calculations was inappropriate here. However, because the small lesion had substantially higher uptake than other tissues in each of the images, its location could be demarcated by setting a threshold. For each image, the target was defined as pixels having values greater than 60% of the maximum pixel value for that image. To exclude uptake erroneously assigned to regions known to be outside the body, a minimum threshold was set (5% of the maximum pixel value). The remaining nonzero pixels defined the background. The target-to-background ratio was calculated as the mean of the target pixel values divided by the mean of the background pixel values. A more intense, localized uptake would have a higher target-to-background ratio.

## 3. RESULTS

**Figure 5:**
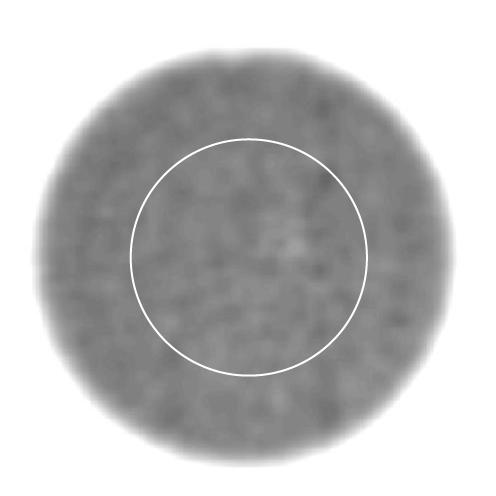
Transaxial 3D brain-mode PET image of a slice through the 9.0 cm diameter phantom cup. The 5.0 cm diameter ROI (white circle) was used to calculate the variance as a measure of image noise since it was known to contain a homogeneous distribution of ^18^F-FDG solution.

**Figure 6:**
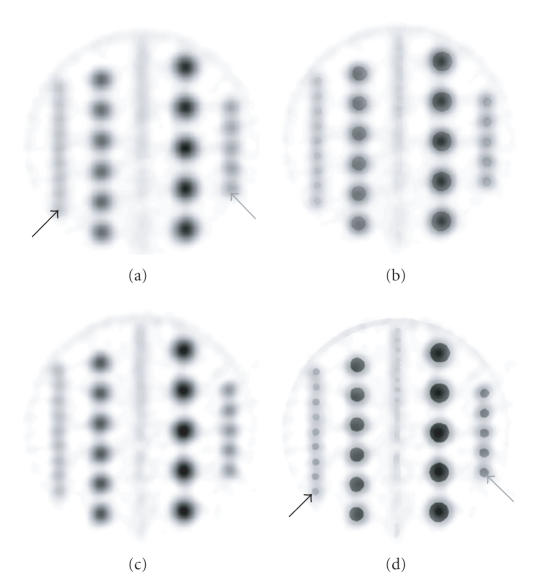
Transaxial PET images through the phantom disk using 3D brain-mode acquisition. (a) Standard processing. The nine hotspots in the row (black arrow) along the left are 3 mm in diameter and the five largest hotspots are 8 mm (gray arrow). (b) HCT result. (c) Super-resolution result. (d) Super-resolution/HCT result has the greatest contrast. The 3 mm sources (black arrow) are more clearly resolved than in the standard image. The 8 mm sources (gray arrow) show sharper edges than in the standard image.

In order to establish that the phantom images had the same noise level, a transaxial slice adjacent to the polycarbonate disk was selected and an ROI used to measure noise was chosen in a region of homogeneous ^18^F-FDG uptake (the white circle in Figure 5). To maintain a variance of 10.6 ± 0.1 kBq^2^/mL^2^ in this ROI, the standard acquisitions were smoothed with a 1.8 mm FWHM Gaussian filter (equivalent to 15 HCT iterations; see (6)) and the super-resolution results were smoothed with a 3.0 mm FWHM Gaussian filter (equivalent to 41 HCT iterations). These filters were also applied on the transaxial images through the polycarbonate disk showing the features of interest (Figure 6).

**Figure 7:**
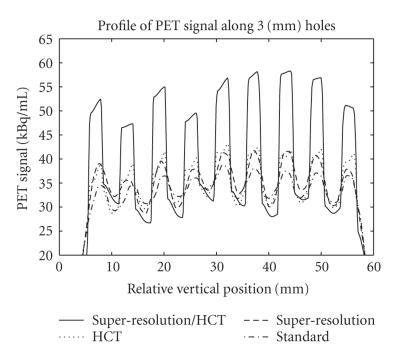
A plot of pixel values through the 3 mm features of the phantom images in Figure 6. The super-resolution (dashed line) and HCT (dotted) profiles give better contrast than the standard method (dashed and dotted). The combination of HCT and super-resolution gives the best contrast (solid black).

In the phantom trial (Table 2), the super-resolution technique improved the concentration ratios of the 3 mm, 4 mm, 6 mm, and 8 mm features from an average of 1.9 (range: 1.1–2.9) for the standard acquisition to an average of 2.1 (range: 1.2–3.3). HCT filtering also improved the standard contrast ratios to an average of 2.1 (range: 1.3–3.1). Using the combined acquisition and processing technique of super-resolution and HCT, the PET contrast ratios were the highest (average: 2.8, range: 1.6–4.3). Using the super-resolution/HCT technique, 3 mm ^18^F-FDG sources were more clearly resolved (Figure 6) than the standard image and the edges of the sources were more delineated. A plot of pixel value profiles through the 3 mm features of the phantom (Figure 7) shows that the super-resolution profile (dashed line) and the HCT profile (dotted) both gave moderately better contrast than the standard method (dashed and dotted). The combination of HCT and super-resolution gave the best contrast of all the methods (Figure 7, solid black line).

The efficacy of including a Gaussian blur kernel in the super-resolution processing [14] was checked by measuring the PSF in the axial direction (2D whole-body mode and 3D brain mode) and transaxial directions (3D brain mode). In each type of image, the “point source” was provided by a cross section through a single 1 mm hole of the phantom which had been filled with ^18^F-FDG and capped. Table 3 shows that, using the same data, the inclusion of a Gaussian blur kernel improved the resolution by reducing the FWHM of the PSFs by a difference of 0.1 mm to 0.2 mm compared to previously reported results [9]. The value of the blur kernel used for Table 3 was set to 3.0 mm since this minimized the FWHM of the “point source.”

In the 2D whole-body mode, when the number of axial shifts was decreased from 4 POVs (with 1 mm shifts) to 2 POVs (with 2 mm shifts), the axial resolution was degraded from 4.0 mm to 4.3 mm as measured by the FWHM of the axial PSF. The axial resolution of the 2D whole-body case did not improve when 8 POVs with 0.5 mm shifts were used; the FWHM of the axial PSF remained at 4.0 mm.

**Figure 8:**
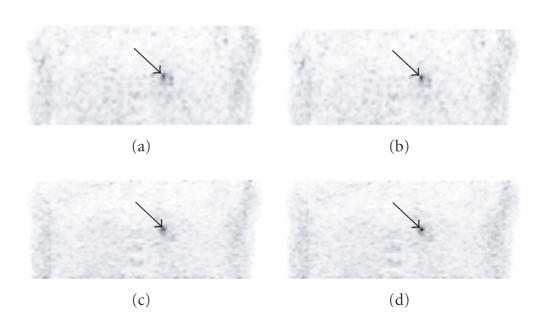
Coronal PET images of the patient through the pulmonary lesion. The black arrow marks the small lesion of interest. (a) Standard 2D whole-body mode acquisition. (b) HCT. The edge of the ^18^F-FDG uptake is more delineated than in the standard image. (c) Super-resolution. The uptake is more localized than in the standard image. (d) Super-resolution and HCT. The uptake is the most localized in this image.

For the patient study in which the super-resolution acquisition time was the same as that of the standard (4 min total), the lesion of interest could not be resolved due to the low number of counts in each POV. By using a 4 min acquisition time for each POV (a total of 16 min), the super-resolution method clearly resolved the lesion as shown in Figure 8(a). In Figure 8, the filters were selected to achieve the same level of noise in the PET images. By smoothing the images with a 3.0 mm FWHM Gaussian filter (10 HCT iterations for the 0.5 mm × 0.5 mm pixel size; see (6)) a variance of 0.36 + 0.01 kBq^2^/mL^2^ was maintained in the coronal images excluding a 15 mm diameter circular ROI around the lesion of interest. Table 4 shows that the lesion target-to-background ratios were higher with super-resolution (except for the sagittal image) when compared to the ratios for the standard images. The application of HCT further increased the target-to-background ratios. For the super-resolution acquisition that was processed with HCT, the target-to-background ratios were the highest. They improved to an average of 8.0 (range: 7.7–8.3) when compared to an average of 6.1 (range: 5.5–6.6) for the standard image. Sharper edges and more localized uptake were also depicted in the patient reconstructions using the combination super-resolution and HCT techniques when compared to the other images (Figure 8).

## 4. DISCUSSION

The super-resolution acquisition and reconstruction meets the goal of obtaining higher resolution in the PET acquisition. Super-resolution has been reported to improve the axial resolution by 9% to 52% compared to a standard acquisition and by 14% to 16% compared to merely interleaving the acquired slices to the appropriate axial location [9]. As described above, modifying the Irani and Peleg method [8] to include a 3.0 mm blur kernel improves these results by a further 2% to 4% (Table 3), using the same data sets. Similarly, in the 3D brain-mode transaxial images, super-resolution has been reported to improve the resolution by at least 12% [9] and the modified method used here improves that result by a further 2%. The improved resolution due to the super-resolution technique compared to a standard acquisition is evident in the phantom image (Figure 6), in a pixel plot through its 3 mm features, and in the improved contrast ratios (Table 2). This improvement due to the super-resolution acquisition and processing holds true even when the super-resolution results require more smoothing than the standard images to achieve the same level of image noise, as in the phantom case.

In the phantom trial (Figure 6), the application of HCT filtering, as an algorithm for the fusion of PET and CT data, improves contrast ratios by an average of 14% (range: 7–18%) when compared to the standard Gaussian method (Table 2). This is similar to the improvement provided by super-resolution alone (average: 13%, range: 9–15%) and the pixel profiles through the 3 mm phantom features using these two methods roughly match (Figure 7). The application of both methods in tandem provides superior contrast ratios: an average of 54% (range: 45–69%) better than the standard processing method for images with the same level of noise. This increase in contrast is a combination of the reduction of partial volume effects provided by super-resolution [9] and the retention of uptake within established borders when the image is smoothed with HCT. Small features are most evident in the super-resolution/HCT image (Figure 6(d)) and pixel profile (Figure 7) when compared to the other three processing methods.

Although the improvement in the image due to the super-resolution technique and the HCT filtering can be demonstrated with the phantom, the same cannot be said for the patient trial since the true distribution of ^18^F-FDG is unknown. However, in all but the sagittal image, super-resolution improved the lesion's target-to-background ratio (Table 4). HCT improved the target-to-background ratio by an average of 26% (range: 12–38%). The combined super-resolution/HCT procedure was superior and improved the target-to-background ratio by an average of 34% (range: 17–47%). In the super-resolution/HCT PET image, the uptake is more localized and delineated (Figure 8) as would be desired for small tumor detection.

Unlike the phantom case, in terms of acquisition time, the comparison between standard and super-resolution patient PET acquisitions is not one to one. The super-resolution acquisition and reconstruction for the patient required approximately four times the number of counts as the standard images. (The signal of the lesion of interest was lost due to the low-counting statistics when the total acquisitions times were kept the same.) Using four POVs of 4 min each, this super-resolution example demonstrates that these acquisitions are clinically feasible if restricted to one FOV of interest. When the total acquisition times were kept constant (as in the phantom case) the super-resolution data required more smoothing (Gaussian filters of 3.0 mm FWHM or their HCT equivalent) than the standard data (1.8 mm FWHM). In contrast, the super-resolution data for the patient did not require additional smoothing to obtain the same noise level as in the standard images (Gaussian filters of 3.0 mm FWHM or their HCT equivalent were used for both) because of the increased number of counts in the super-resolution case.

The choice of 4 POVs for the super-resolution technique in the patient case is reasonable. Since the automated bed motion readily provides increments of 0.5 mm, conceivably one could acquire 8 POVs for the super-resolution technique. However, at 4 min per POV the resulting long acquisition time may be prohibitive. On the other hand, keeping the total acquisition time constant renders the number of counts per position too low to be useful, as found in the four 1-minute POVs case. In general it could be stated that there is a minimal acquisition time required for each POV in order to obtain useful information. Hence, the number of POVs multiplied by that minimal acquisition time will determine the needed total acquisition time. The number of POVs used and their corresponding acquisition times has yet to be optimized.

It is worth reiterating from [9] that patient motion will further degrade the efficacy of the super-resolution technique because the registration of the POVs should be known to subpixel accuracy. Consequently, brain scans may be more suitable for the clinical application of super-resolution since the head is then firmly fixed and subject to little motion. Also, the application of this technique in the transverse direction would require a method of recording the geometric shifts of the patient in the transaxial plane. Conceivably, one could envision a new type of scanner with a rotating gantry, and perhaps even with some transaxial motion, that would be able to provide super-resolution without moving the patient.

Applying HCT in the axial direction as presented here is suboptimal since the slice thickness of the CT was automatically set by the scanner to be the same as that of standard PET images. However, the CT scanner can potentially provide thinner slice reconstructions. Using such images as the CT input would reduce partial volume effects and potentially further improve the results.

The improvement in resolution due to super-resolution acquisition and reconstruction and the improvement in contrast ratio using HCT filtering come at a considerable increase in computational time when applied together. Compared to standard processing, the super-resolution technique applied to PET increases processing times by a factor of 23 [9] and HCT filtering increases this by a factor of 8 [11]. On the Discovery-LS scanner used, the reconstruction time of AC PET is typically 2 to 3 min per FOV with most of the reconstruction being performed concurrent with a 20-to-30-minute acquisition of 5 to 7 FOVs per patient. Increasing processing times by factors greater than 8 could not be easily accommodated. Because of this prohibitive increase in computer processing time, the clinical application of the combined super-resolution/HCT process would likely need suitable dedicated computer hardware or to be restricted to a suspicious region of interest to avoid spending computational resources sharpening the entire data set.

As an alternative to OSEM, one may consider the use of penalized-likelihood image reconstruction methods, as a complementary process to super-resolution. Penalized-likelihood iterative reconstruction algorithms include a penalty (regularization) term which discourages neighboring pixels from converging to widely disparate values [23]. With such an approach, edge information (obtained from another modality) may be introduced via the regularization term [24] or prior [25], and perhaps could replace the HCT processing stage. A disadvantage of using penalized-likelihood methods for emission tomography is that space-invariant penalties result in high-count regions tending to be smoothed more than low-count regions [26], but methods have been developed to give a more uniform spatial resolution [27]. Although not addressed by this paper, it would be worthwhile to try to achieve a similar improvement in resolution for a given variance by combining the super-resolution method with the penalized-likelihood reconstruction methods.

## 5. CONCLUSION

A new method incorporating two techniques, super-resolution and hybrid computed tomography (HCT), for fusing PET and CT images has been developed and evaluated. A super-resolution acquisition, modified to include a Gaussian blur kernel, has been shown to significantly improve the resolution of the PET acquisition. The feasibility of implementing the method in a clinical PET/CT scanner has been demonstrated by showing higher contrast ratios in a phantom study and higher target-to-background ratios in a small lesion from a patient study for images exhibiting the same level of noise. The resulting reconstructions provide higher resolution metabolic images with delineated edges where corresponding anatomical borders are available.

## Figures and Tables

**Table 1:** Transaxial displacements and rotations from the initial position used in the 3D AC brain-mode acquisition phantom trial.

2.5-minute PET displacement acquisition	Horizontal displacement left (mm)	Vertical displacement up (mm)	Clockwise rotation (degrees)

Initial	0	0	0
Second	2.0	0.5	1.7
Third	5.0	1.2	3.9
Fourth	9.1	2.0	7.2

**Table 2:** Contrast ratios for the PET signals in the 3D AC brainmode acquisition phantom trial.

Image type	3 mm	4 mm	6 mm	8 mm
	holes	holes	holes	holes

Standard	1.1	1.3	2.1	2.9
Super-resolution	1.2	1.5	2.4	3.3
HCT	1.3	1.5	2.4	3.1
HCT and super-resolution	1.6	2.2	3.2	4.3

**Table 3:** Super-resolution point spread function FWHM values for phantom trials.

Acquisition mode	Axis	Blur kernel of 1 pixel^(a)^ (mm)	Gaussian blur kernel of 3.0 mm FWHM (mm)

2D whole body	Axial	4.1	4.0
3D brain	Axial	4.8	4.6
3D brain	Radial	4.4	4.3
3D brain	Tangential	4.3	4.2

^(a)^Previously reported [9].

**Table 4:** Lesion target-to-background ratios for the PET signals in the 2D whole-body mode acquisition patient trial.

Image type	Transaxial	Coronal	Sagittal	Average

Standard filter	5.5	6.0	6.6	6.1
(3.0 mm FWHM)				
Super-resolution	6.3	6.3	5.9	6.2
HCT	7.6	7.7	7.4	7.6
HCT and super-resolution	8.1	8.3	7.7	8.0
